# Structural purity of magnetite nanoparticles in magnetotactic bacteria

**DOI:** 10.1098/rsif.2010.0576

**Published:** 2011-01-19

**Authors:** Anna Fischer, Manuel Schmitz, Barbara Aichmayer, Peter Fratzl, Damien Faivre

**Affiliations:** Department of Biomaterials, Max Planck Institute of Colloids and Interfaces, Science Park Golm, 14424 Potsdam, Germany

**Keywords:** biomineralization, magnetite, magnetotactic bacteria, synchrotron X-ray diffraction, lattice parameter, hierarchical structuring

## Abstract

Magnetosome biomineralization and chain formation in magnetotactic bacteria are two processes that are highly controlled at the cellular level in order to form cellular magnetic dipoles. However, even if the magnetosome chains are well characterized, controversial results about the microstructure of magnetosomes were obtained and its possible influence in the formation of the magnetic dipole is to be specified. For the first time, the microstructure of intracellular magnetosomes was investigated using high-resolution synchrotron X-ray diffraction. Significant differences in the lattice parameter were found between intracellular magnetosomes from cultured magnetotactic bacteria and isolated ones. Through comparison with abiotic control materials of similar size, we show that this difference can be associated with different oxidation states and that the biogenic nanomagnetite is stoichiometric, i.e. structurally pure whereas isolated magnetosomes are slightly oxidized. The hierarchical structuring of the magnetosome chain thus starts with the formation of structurally pure magnetite nanoparticles that in turn might influence the magnetic property of the magnetosome chains.

## 1. Introduction

Biomineralized nanoparticles are as diverse as the functions they fulfil in the multiple organisms in which they are formed [1]. The organisms control the synthesis and organization of hybrid materials to achieve higher functional properties [2]. In magnetotactic bacteria, one of the simplest biomineralizing organisms, the genetic blueprint information is translated into complex inorganic and cellular structures, i.e. the magnetosomes [3,4]. Prokaryotes have developed a genetic apparatus enabling them to synthesize monodisperse crystals of magnetite (Fe_3_O_4_) for effective magnetic orientation [5,6]. Control of crystal size via physico-chemical [7] or genetic [8] means, the associated magnetic properties [9,10] together with the tuning of the magnetic properties, thanks to the addition of given metallic ions [11,12], have attracted a multi-disciplinary interest to the magnetosomes, specifically using them for bio- and nanotechnological applications [13,14].

The magnetosomes are assembled into a linear chain, representing a first level of structural hierarchy at the sub-micrometer scale [15,16]. Moreover, the biomineralization of the intracellular magnetite is controlled to dimensions within the stable single-magnetic-domain size range, representing the second level of hierarchical structuring at the nanometre-scale. In such a configuration, the total magnetic dipole moment is the sum of the moments of individual particles, thereby generating an optimized configuration for function and applications [5,13,17,18]. However, long-standing debates concerning the structural perfection of magnetosomes at the ångström level and the possible presence of maghemite (α-Fe_2_O_3_) still remain [19–22]. Specifically, the limited precision of electron diffraction with respect to lattice parameter determination prevented detailed quantitative comparison between different biogenic magnetites and abiotic magnetites. However, as shown for biogenic aragonite [23] and calcite [24,25]—when compared with analogous abiotic crystals—anisotropic lattice distortions could be revealed by high-resolution X-ray diffraction (XRD), justifying the need for precise characterization of the microstructure of biogenic magnetite. High-resolution powder XRD was thus used to measure lattice parameters of nano-sized biogenic magnetite at the BESSY II synchrotron [26]. Whole cells of *Magnetospirillum gryphiswaldense* (strain MSR-1), *Magnetospirillum magneticum* (strain AMB-1) and Δ*mamGFDC*, a deletion mutant of *M. gryphiswaldense* with altered crystallite size [8], as well as isolated and detergent-treated MSR-1 magnetosomes [27] were measured. Abiotic reference magnetite and maghemite were used for comparison.

## 2. Material and methods

### 2.1. Biological and inorganic samples

*M. gryphiswaldense* (MSR-1) [28] and *M. magneticum* (AMB-1) [29] cells were used throughout the experiments. AMB-1 and MSR-1 strains were chosen because they are the most widely used model organisms of magnetotactic bacteria, partly because they have been sequenced and their genetic systems have been established [4]. Δ*mamGFDC* was provided by D. Schüler (LMU Munich, Microbiology department) and was used to determine if size effects on lattice parameter are present. All strains were cultured in the rubber cap-sealed culture tubes under microaerobic conditions in MSR-1 standard media [27]. Bacterial growth was determined by measuring the optical density (OD) at 565 nm (Shimadzu UV-1201V spectrophotometer). The magnetic orientation of cells was determined by optical measurements (*C*_mag_) [30]. The tubes were inoculated with 1 ml of a respective pre-culture (OD ≈ 0.4; *C*_mag_ ≈ 0.8) and incubated at 28°C and 100 r.p.m. for 24 h.

Magnetosome isolation and treatment were realized as described in literature [27]. Isolated magnetosomes with membrane are denoted as MAG + MM and without membrane, after sodium dodecyl sulphate (SDS) treatment, as MAG−MM. The synthetic magnetite (MGT) and maghemite (MGH) samples were provided by the German Federal Institute for Materials Research and Testing (BAM).

### 2.2. Transmission electron microscopy

About 1 ml of the cell suspension was used for grid preparation. The probes were centrifuged at 14 000 r.p.m for 5 min and resuspended in 100 µl of medium. A Cu grid with an amorphous carbon support film was deposited on a drop of the preparation and let for about 10 min for adsorption. The grids were subsequently removed, washed with deionized water and dried with filter paper. Transmission electron micrographs were acquired on a Zeiss EM Omega 912× at an acceleration voltage of 120 kV. Particle dimensions were analysed using standard analytical software for processing digital electron microscope images (ImageJ) as described in the literature [31]. Briefly, a watershed segmentation was applied when enough contrast was given, and the particles were approximated by ellipses. However, when no clear segmentation could be obtained, the particles were measured manually, with two axes. As the average crystallite size of the sample matters for size effects in XRD, about 900 particles (about 30 cells) were counted to determine the average size. The composition of the materials was determined by energy dispersive X-ray spectroscopy (EDX) on a transmission electron microscope from Zeiss LIBRA 200 operated at 200 kV equipped with an EDX detector from Thermo Fisher operated with the ‘System 6’ software.

Electron tomography was performed on the same Zeiss LIBRA 200 microscope. For this purpose gold marker Copper grids were employed. A tilt series from −76° to 76° with 1° steps was obtained. The reconstruction was performed with IMOD (http://bio3d.colorado.edu/imod/) and the visualization with VG Studio MAX 1.2.

### 2.3. Synchrotron X-ray diffraction

XRD measurements were performed at the µ-spot beamline at the BESSY II synchrotron radiation facility (Helmholtz-Zentrum Berlin (HZB), Germany) [26], in transmission geometry, with an energy of 15 keV (*λ* = 0.82656 Å), defined by a silicon (111) double-crystal monochromator and a beam size of 30 µm. Two-dimensional scattering patterns were collected using a MarMosaic 225 charge-coupled device-based area detector (Rayonix, USA). Prior to the measurements, the cells were centrifuged at 4°C (8000 r.p.m, 10 min). The resulting pellets were carefully washed (three times) with Millipore water in order to remove any buffer traces from the medium. Concentrated cell suspensions were then deposited on ultra-thin Kapton foil (7 µm thick) and let to dry. The Kapton foil, which exhibits a weak scattering background, had been clamped to a specially designed multiple sample holder, providing a flat separate window for each sample suspension. For the calibration of the sample to detector distance, indispensable for high-accuracy lattice parameter measurements, each sample was mixed prior to drying with 5 per cent of α-quartz (NIST, standard Reference Material 1878a) as an internal quantitative XRD standard. Each sample was measured at three different positions on the sample holder window to ensure good statistics and reproducibility of the measurements. For each two-dimensional diffraction pattern, beam centre and tilt corrections were performed using Fit2D (http://www.esrf.eu/computing/scientific/FIT2D/) and refined by a home-developed beam-centre determination routine. The two-dimensional pattern was integrated to a one-dimensional diffractogram (Intensity versus *q*, *q* = 4*π* sin (*θ*)/*λ*, where *λ* is the wavelength and 2*θ* corresponds to the scattering angle) followed by calibration to the (101) peak position of the α-quartz (*q*_101_ = 18.7910 nm^−1^). After baseline correction, a pseudo-Voigt function was used for fitting to determine peak positions.

Lattice parameters were calculated based on the assumption of a cubic space lattice (*a* = *b* = *c*; *α* = *β* = *γ* = 90°; *a* = *d*_*hkl*_/(*h*^2^ + *k*^2^ + *l*^2^)^1/2^) for all diffraction peaks with considerable intensity greater than 20 counts and well-defined peak shapes. An average lattice parameter was calculated from the obtained values for each sample and the error of the lattice parameter was calculated as standard deviation, as shown later in table 1 and figure 3. Particle sizes were estimated from the peak width after correcting for instrumental broadening effects. Approximating the Bragg peaks by Gaussian profiles, the peak broadening *W*_tot_ (full width at half maximum in *q*-space) can be written as follows [26]:2.1
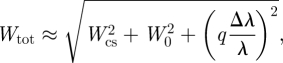
where *W*_cs_ corresponds to the crystal size related broadening and *W*_0_ depends on the instrumental set-up (beam divergence, detector point spread function and distance). The finite wavelength spread Δ*λ*/*λ* leads to a *q*-dependent instrumental broadening. Additional *q*-dependent contributions owing to possible microstrain fluctuations were not observed and could hence be neglected. If a polycrystalline sample comprising sufficiently large crystals is considered (such as the used NIST standard and the synthetic magnetite and maghemite), the sample-related peak broadening is almost zero and a regression analysis of the *q*-dependent broadening allows for determining both, *W*_0_ and Δ*λ*/*λ*. The synthetic magnetite sample was used to determine the instrumental broadening, as no differences in *W*_tot_between the α-NIST powder and the synthetic magnetite were observed. The obtained values of 0.10266 nm^−1^ and 0.00167, for *W*_0_ and Δ*λ*/*λ*, respectively, are in good agreement with the beamline performance for a sample to detector distance of approximately 140 mm, a 30 µm beam-defining pinhole and the energy resolution of the Si (111) monochromator. Finally, the particle size (PS) was estimated from *W*_cs_ using Scherrer's equation:2.2
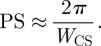

**Table 1.** Lattice parameter *a* determined for each diffraction peak, averaged lattice parameter *a*_a_, particle size, PS, determined by XRD and by TEM (maximum of the distribution) for each sample. Difference in magnetosome dimensions between particles in the bacteria and isolated ones are due to the isolation process.

sample	(220)	(311)	(400)	(422)	(511)	(440)	*a*_a_ (Å)	standard devation (Å)	PS_XRD_ (nm)	PS_TEM_ (nm)
MSR-1	8.3983	8.3981	8.3979	8.3975	8.3971	8.3968	8.3968	0.0009	44	40
	8.3958	8.3971	8.3968	8.3975	8.3963	8.3957				
	8.3975	8.3967	8.3960	8.3962	8.3954	8.3953				
AMB	8.3969	8.3972	—	8.3966	8.3957	8.3956	8.3958	0.0007	44	45
	8.3949	8.3959	8.3956	8.3958	8.3951	8.3948				
	8.3948	8.3965	8.3965	8.3963	8.3955	8.3953				
Δ*mamGFDC*	8.3960	8.3973	8.3955	8.3967	8.3954	8.3957	8.3965	0.0013	31	25
	—	8.3957	—	—	8.3965	8.3942				
	8.3979	8.3987	8.3973	8.3974	—	8.3968				
MAG + MM	8.3886	8.3892	8.3889	8.3888	8.3884	8.3881	8.3875	0.0009	33	35
	8.3868	8.3876	8.3872	8.3872	8.3868	8.3867				
	8.3868	8.3874	8.3871	8.3870	8.3867	8.3866				
MAG−MM	8.3673	8.3679	8.3676	8.3675	8.3672	8.3671	8.3687	0.0014	33	35
	8.3701	8.3706	8.3702	8.3699	8.3696	8.3694				
	—	—	—	—	—	—				
MGT	8.3916	8.3921	8.3915	8.3912	8.3911	8.3911	8.3907	0.0009	—	300–700
	8.3896	8.3903	8.3904	8.3892	8.3894	8.3890				
	8.3910	8.3921	8.3913	8.3905	8.3907	8.3904				
MGH	8.3458	8.3467	8.3466	8.3459	8.3464	8.3453	8.3470	0.0010	—	100–200
	8.3467	8.3475	8.3475	8.3467	8.3472	8.3460				
	8.3478	8.3486	8.3486	8.3478	8.3481	8.3469				

## 3. Results and discussion

**Figure 1.**
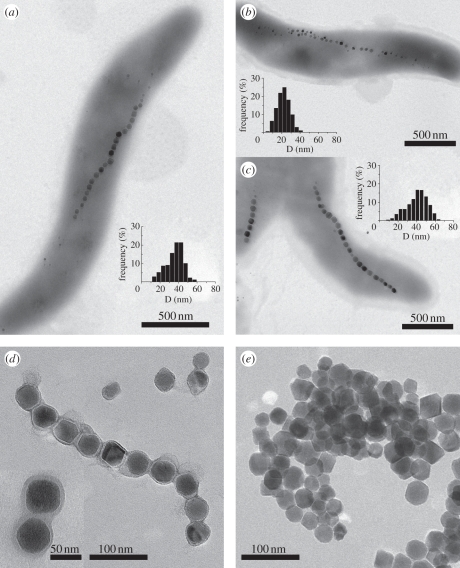
TEM micrographs and particle size distribution of magnetite particles in magnetotactic bacteria: (*a*) MSR-1, (*b*) Δ*mamGFDC* and (*c*) AMB-1 cell (scale bar, 500 nm). (*d*) and (*e*) show, respectively, isolated magnetosomes from MSR-1 with membrane, as highlighted in the inset (scale bar, 50 nm) and without membrane (scale bar, 50 nm). Both samples were stained with uranyl acetate prior measurement.

**Figure 2.**
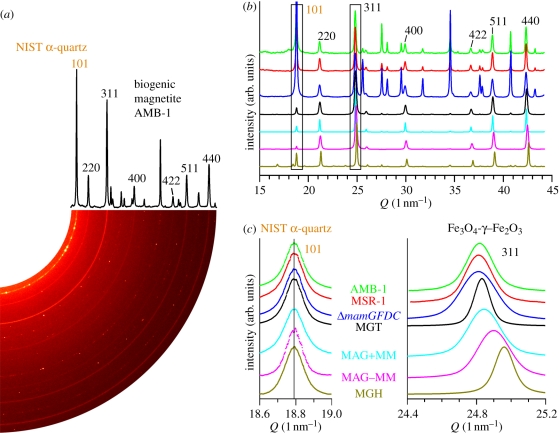
(*a*) Two-dimensional and one-dimensional XRD pattern of the biogenic AMB-1 sample with NIST standard. Only the peaks used for data analysis are indexed. (*b*) Integrated one-dimensional X-ray diffractograms for all measured samples. All indexed diffraction peaks correspond to magnetite (or maghemite), while the remaining diffraction peaks belong to the α-quartz standard peaks. The (311) MGT (MGH) and (101) α-quartz peak are highlighted (boxes). (*c*) Enlargement of the (101) α-quartz diffraction peak and of the (311) diffraction peak of MGT. Depicted are MGT in whole cells of strains AMB-1 (green), MSR-1 (red) and Δ*mamGFDC* (blue), isolated (turquoise) and treated (rose) magnetosomes, and reference synthetic MGT (black) and MGH (brown).

**Figure 3.**
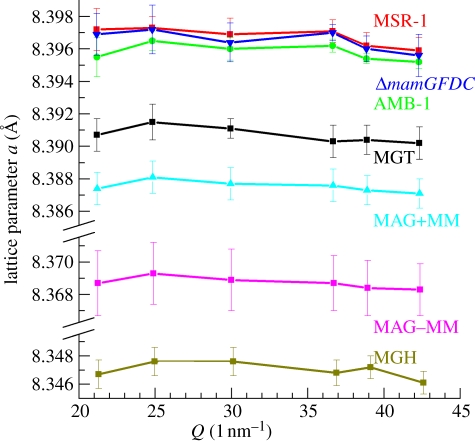
Calculated lattice parameters with standard deviations for biotic and abiotic samples for different magnetite and maghemite diffraction peaks. Same colours and samples as in figure 2.

Typical transmission electron microscopy (TEM) images of the different strains of magnetotactic bacteria and the associated crystal size distribution are shown in figure 1. A two-dimensional diffraction pattern with the corresponding one-dimensional diffractogram obtained for the AMB-1 sample is shown in figure 2*a*. One-dimensional diffractograms of all samples are shown in figure 2*b* with an enlargement in figure 2*c* of the most intense (311) and (101) reflections of, respectively, magnetite/maghemite and α-quartz, the latter being used as internal standard (supporting info). For all samples, the diffraction patterns could be indexed according to magnetite (respectively, maghemite), cubic unit cell (space group *Fd*3*m*). The lattice parameter, *a*, was calculated by fitting the angular positions of the measured Bragg peaks. The *a*-values, extracted from individual diffraction peak positions, as well as the averaged lattice parameter *a*_a_ are summarized in table 1 and figure 3.

### 3.1. Lattice parameters

Our measurements reveal that the measured lattice parameters are similar for the bacterial samples (*a*_a_ _MSR-1_ = 8.3968 ± 0.0009 Å; *a*_a_ _AMB_ = 8.3958 ± 0.0007 Å and *a*_a_ _Δ*mamGFDC*_ = 8.3965 ± 0.0013 Å) and comparable with the literature value of stoichiometric MGT (*a* = 8.3969 ± 0.0008 Å) [32]. In contrast, the magnetosomes isolated from the bacteria, but still protected by their membrane, exhibited a smaller lattice parameter (*a*_a_ _MAG + MM_ = 8.3875 ± 0.0009 Å), which is comparable to that of our reference synthetic MGT (*a*_a_ _MGT_ = 8.3907 ± 0.0009 Å). Finally, the treated magnetosomes, also lacking the magnetosome membrane, presented an even smaller lattice parameter (*a*_a MAG − MM_ = 8.3687 ± 0.0014 Å), representing an intermediate step to MGH (*a*_a MGH_ = 8.3470 ± 0.0010 Å).

### 3.2. Origin of the different lattice parameters

Typical explanations for such lattice parameter variations include the surface stress effect in nanoparticles [33], changes in composition [34] or the presence of intracrystalline proteins in ceramic crystallites [24]. The lattice parameter of the investigated biogenic magnetite did not show any dependence on crystallite size. Indeed, correcting the diffraction data for instrumental broadening and using Scherreŕs equation to calculate the size from corrected peak broadening [26], particle dimensions of 44, 44 and 31 nm were determined for MSR-1, AMB-1 and Δ*mamGFDC*, respectively (table 1), being in good agreement with TEM measurements (figure 1 and table 1) and literature values [4]. Moreover, only particles smaller than 20 nm typically exhibit a significant surface stress effect on the lattice parameters [33].

Protein inclusions in the mineral could also be excluded as magnetosome and biomacromolecules have similar dimensions. Moreover, only surface-bound proteins and no protein inclusions within the mineral were evidenced in magnetosomes [35]. Hence, only variations in the composition of the samples, such as chemical impurities and stoichiometry, could explain the observed differences in lattice parameters. As the biogenic samples were all grown in the same media, no change of chemical composition in terms of impurities were expected between the different biogenic samples, even after magnetosome isolation. However, the composition of the samples was investigated to compare the biogenic and the abiotic sample. Based on EDX measurements (table 2), a potential effect of chemical impurities on lattice parameters could be excluded, as the biogenic and abiotic samples exhibited similar compositions. A total amount of impurities of 0.7 at% (Ti, Si, Cl) in the biogenic and 0.5 at% (Si, Mn) in the abiotic sample were measured. It is unclear if the impurities could really be incorporated in the mineral, or would originate from outlying grid contamination owing to sample preparation. However, even when considering that the impurities were incorporated in the mineral, the observed differences in lattice parameter would not be obtained. Indeed, while Mn and Ti-doping increase the spinel lattice [34], Si doping [36] decreases it, the later case being relevant in our observations here. In both cases, the impurity amount in the samples is too small to explain the observed shifts. For the Si substitution, a substitution degree of *x* = 0.09 in Fe_(3−*x*)_Si_x_O_4_—equivalent to an Si amount of approximately 4 at%—would be necessary to induce a lattice parameter decrease of 2 × 10^−3^ Å [36], as observed here between the biogenic and abiotic samples. So to sum up, only stoichiometric effects can explain the observed difference.

**Table 2.** Chemical composition of the biotic and abiotic magnetite sample determined by EDX measurements.

biogenic magnetite		abiotic magnetite	
element	concentration (at%)	element	concentration (at%)
O	56.8 ± 0.5	O	57.6 ± 0.5
Fe	41.4 ± 0.2	Fe	41.7 ± 0.2
Si	0.5 ± 0.1	Si	0.3 ± 0.1
Ti	0.1 ± 0.05	Mn	0.2 ± 0.05
Cl	0.1 ± 0.05		

Oxidation of magnetite to maghemite easily takes place at low temperatures by solid-state reaction via intermediate *z*-oxidation state [37]:3.1
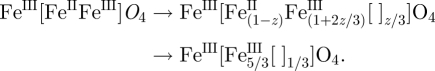


**Figure 4.**
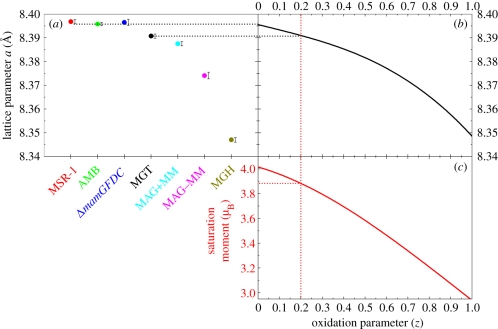
(*a*) The lattice parameters measured for the samples are reported (same colours and samples as in figure 2). In (*b*) the oxidation parameter *z* of each sample can be determined based on the fit *a*(*z*) = 8.3956 − 0.0224*z* + 0.0026*z*^2^ − 0.0273*z*^3^, *R*^2^ = 0.990) of the experimental data of Dunlop & Özdemir [37]. Focusing on the biogenic (*z* = 0.00) and abiotic (*z* = 0.21) MGT samples, the saturation moment *M*_s_ can be seen in (*c*) based on their oxidation parameter and on the fit *M*_s_ (*z*) = 4.0285 − 0.6983*z* − 0.3961*z*^2^, *R*^2^ = 0.998) of the experimental data of Dunlop & Özdemir [37]. The biogenic magnetite samples thereby exhibit *M*_s_ = 4.03 µ_B_ whereas the abiotic has a calculated saturation moment of 3.86 µ_B_.

While the inverse spinel structure and the face-centred cubic unit cell are conserved, maghemitization results in a lattice parameter decrease [37]. This reduction is induced by the creation of vacancies in the iron lattice and the change in Goldschmidt radius from 0.83 to 0.67 Å, as Fe(II) is oxidized to Fe(III) [37]. The lattice parameter of the bacterial magnetite fits perfectly with stoichiometric magnetite [32], whereas that of the reference sample reveals slight oxidation (figure 4). This result is not surprising since Fe(II) can easily be oxidized to Fe(III) under environmental conditions. With a lattice parameter of *a*_a MGT_ = 8.3907 Å, and a third-order polynomial (best) fit of the literature experimental *a*(*z*) plot [37] (figure 3), an oxidation state of *z* = 0.21 can be estimated for the reference abiotic magnetite that in our opinion depicted the equilibrium state of magnetite nanoparticles under atmospheric conditions.

### 3.3. Implications for magnetite formation in magnetotactic bacteria

The synthesis of magnetite typically requires physico-chemical conditions that are basic and reductive (the thermodynamic stability domain for MGT is centred around pH ≈ 10 and Eh ≈ −0.5 V) [4]. These conditions should be encountered within the magnetosome organelles in order to form the structurally pure magnetite we measured. However, the physico-chemical conditions are different in the growth medium (pH ≈ 7 and Eh ≈ 0 V). The bacteria thus most probably play an active role in synthesizing and stabilizing their magnetic inclusions. This is confirmed by the fact that the bacterial protection is no longer guaranteed for isolated magnetosomes. In this case, the lattice parameter decreases to a value similar to that of the reference magnetite, clearly evidencing that isolated magnetosomes start to oxidize. A further dramatic lattice parameter decreases owing to oxidation is induced by hot SDS, which destroys the protective magnetosome membrane and leads to an even more pronounced oxidation.

### 3.4. Putative role of stoichiometric magnetite in bacteria

Stoichiometric magnetite is ferrimagnetic with the highest magnetic moment (*ca* 4 µ_B_) when compared with other iron oxide [37]. Maghemitization diminishes the resulting saturation moment to a value of *ca* 3 µ_B_ for maghemite [37]. By fitting the literature experimental saturation moment *M*_s_(*z*) [37] with a second-order polynomial function (best fit), a saturation moment of 3.86 µ_B_ is obtained for the reference abiotic magnetite (*z* = 0.21, figure 3). This corresponds to a loss of saturation moment of 4.1 per cent compared with stoichiometric magnetite formed by the bacteria. We, therefore, speculate that magnetotactic bacteria might optimize their functionality at the ångström level by facing the challenging task of synthesizing and maintaining the structure of stoichiometric magnetite.

## 4. Conclusion

**Figure 5.**
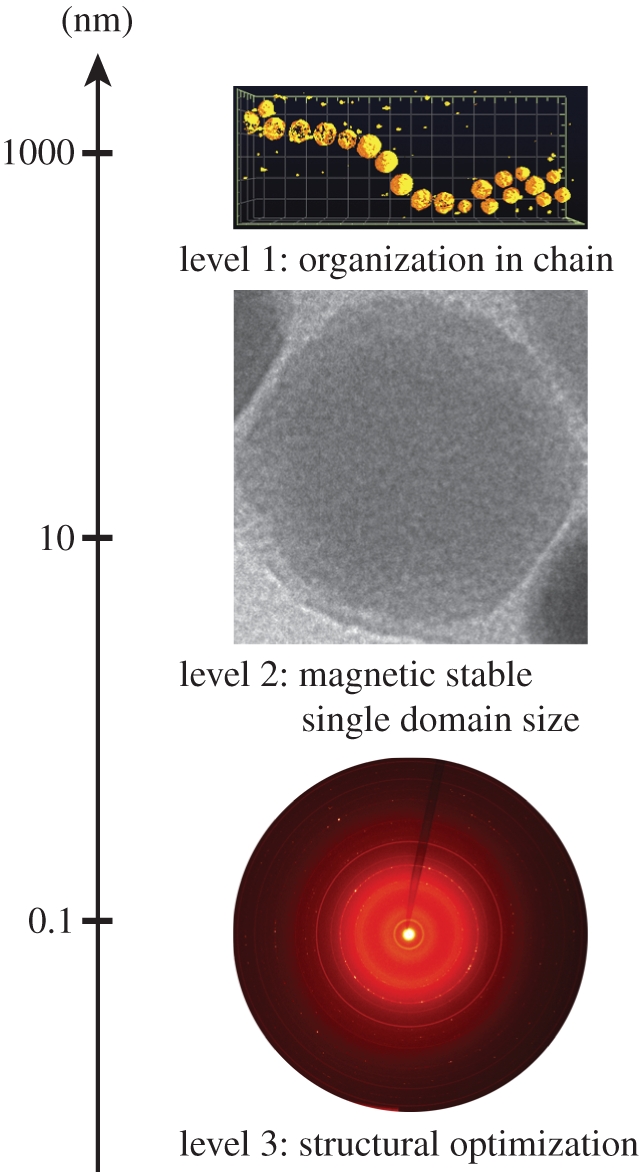
Overview of the different levels of hierarchy encountered in magnetotactic bacteria with respect to their length scale. Level 1: electron tomography reconstruction of a MSR-1 magnetosome chain of about 1 µm length. Level 2: TEM image of an isolated MSR-1 magnetosome, the width of the image represents 50 nm. Level 3: two-dimensional diffractogram of whole cells AMB-1, the lattice parameter is optimized at a sub-nanometre scale. This final new level was identified in this study.

We have studied the structure of magnetite nanoparticles from biogenic and abiotic origin by high-resolution XRD. We measured a significant difference in lattice parameter between the biological and the synthetic materials and between isolated and non-treated biological materials. We could show that this difference was associated with different oxidation state and particularly that the original and non-treated biogenic nanomagnetite is stoichiometric, i.e. structurally pure. We hypothesized that this can only be performed if the bacteria actively generate optimal physico-chemical conditions within their organelles. The cells are able to biomineralize stoichiometric magnetite at room temperature, whereas stoichiometric inorganic nanomagnetite is unstable when not protected from oxidation. Moreover, the difference observed between the biological and synthetic samples at room temperature of only 0.067 per cent in the lattice parameter depicts a change of 4.1 per cent in the respective magnetic moment. We thus speculated that the exceptional magnetic properties of the magnetotactic bacteria arose not only from the successive hierarchical level of magnetosome dimension and organization into chains but also from the atomic structure of the magnetic biological material (figure 5). This hierarchical structuring is a striking example of nature's ability of structure–function optimization. In addition, the difference in lattice parameter measured between isolated and non-treated biological materials might help explain controversial results of bulk magnetic studies that found anomalous behaviour for bacterial magnetosomes. We think that it would be of interest to apply such high-resolution XRD technique to study the magnetosomes doped with metals other than iron and other further genetic modification of the magnetosomes that might impact the biomineralization process. We believe that the study of the structural perfection of unique nanometre-scaled biological materials and the underlying mechanisms of their synthesis will aid in the design of advanced magnetic materials conceptually inspired by the natural system.
